# Pharmacological activities of chemically characterized essential oils from *Haplophyllum tuberculatum* (Forssk.)

**DOI:** 10.3389/fchem.2023.1251449

**Published:** 2023-10-06

**Authors:** Abdelkrim Agour, Ibrahim Mssillou, Aimad Allali, Mohamed Chebaibi, Youness El Abdali, Azeddin El Barnossi, Yousef A. Bin Jardan, Gezahign Fentahun Wondmie, Hiba-Allah Nafidi, Mohammed Bourhia, Amina Bari, Badiaa Lyoussi, Elhoussine Derwich

**Affiliations:** ^1^ Laboratory of Natural Substances, Pharmacology, Environment, Modeling, Health, and Quality of Life, Faculty of Sciences Dhar El Mahraz, University Sidi Mohamed Ben Abdellah, Fez, Morocco; ^2^ Ministry of Health and Social Protection, Higher Institute of Nursing Professions and Health Techniques, Taza, Morocco; ^3^ Ministry of Health and Social Protection, Higher Institute of Nursing Professions and Health Techniques, Fez, Morocco; ^4^ Biomedical and Translational Research Laboratory, Faculty of Medicine and Pharmacy of the Fez, University of Sidi Mohamed Ben Abdellah, Fez, Morocco; ^5^ Laboratory of Biotechnology, Environment, Agrifood, and Health, Faculty of Sciences Dhar El Mahraz, University of Sidi Mohamed Ben Abdellah, Fez, Morocco; ^6^ Department of Pharmaceutics, College of Pharmacy, King Saud University, Riyadh, Saudi Arabia; ^7^ Department of Biology, College of Science, Bahir Dar University, Bahir Dar, Ethiopia; ^8^ Department of Food Science, Faculty of Agricultural and Food Science, Laval University, Quebec City, QC, Canada; ^9^ Department of Chemistry and Biochemistry, Faculty of Medicine and Pharmacy, Ibn Zohr University, Laayoune, Morocco

**Keywords:** *Callosobruchus maculatus*, *Haplophyllum tuberculatum*, *in-silico*, mold, stored seeds

## Abstract

The present work aimed at characterizing the phytochemical composition of *Haplophyllum tuberculatum* essential oil (HTEO), assessing its antifungal activity against various fungal strains, evaluating its insecticidal and repulsive properties against *Callosobruchus maculatus*, and determine its antioxidant capacity. To this end, Gas chromatography-mass spectrometry analysis detected 34 compounds in HTEO, with *β*-Caryophyllene being the major constituent (36.94%). HTEO demonstrated predominantly modest antifungal effects, however, it sustains notable activity, particularly against *Aspergillus flavus*, with an inhibition rate of 76.50% ± 0.60%. Minimum inhibitory concentrations ranged from 20.53 ± 5.08 to 76.26 ± 5.08 mg/mL, effectively inhibiting fungal growth. Furthermore, the antifungal, and antioxidant activities of HTEO were evaluated *in silico* against the proteins *Aspergillus flavus* FAD glucose dehydrogenase, and beta-1,4-endoglucanase from *Aspergillus niger*, NAD(P)H Oxidase. Moreover, HTEO displayed strong insecticidal activity against *C. maculatus*, with contact and inhalation tests yielding LC_50_ values of 30.66 and 40.28 μL/100g, respectively, after 24 h of exposure. A dose of 5 μL/100g significantly reduced oviposition (48.85%) and inhibited emergence (45.15%) compared to the control group. Additionally, HTEO exhibited a high total antioxidant capacity of 758.34 mg AAE/g EO, highlighting its antioxidant potential. *Insilico* results showed that the antifungal activity of HTEO is mostly attributed to *γ*-Cadinene and p-Cymen-7-ol, while antioxidant is attributed to *α*-Terpinyl isobutyrate displayed. Overall, HTEO offers a sustainable and environmentally friendly alternative to synthetic products used to manage diseases.

## 1 Introduction

Medicinal plants have been utilized across the world to treat and prevent diseases due to their wide range of therapeutic benefits and their cost-effectiveness (Petrovska, 2012). Notably, these plants are known to possess a panoply of pharmacological properties which also corresponds to the presence of a plethora of bioactive compounds they possess. These bioactive compounds have been noted to reside in different parts of the plants, however, the EO of aromatic plants such as *Haplophyllum tuberculatum* (Forsskal) (*H. tuberculatum*) is recently becoming a cynosure due to their mildly explored therapeutic potentials (Hamdi et al., 2017a). These plants are majorly explored for their pharmacological properties including antioxidant activity, antimicrobial activity, and insecticidal activity among many others (Raissi et al., 2016).

In the context of the exploration of EO of medicinal plants, diseases caused by Fungal including *Aspergillus* and *Fusarium* species have constantly remained at the center of attraction due to their enormous negative impact on agricultural yields worldwide, with these impacts cascading to both animals and humans due to the central role played by agricultural products in the sustaining of the ecosystem (Belhi et al., 2022). Specifically, *Aspergillus flavus* (*A. flavus*) is a pathogen that is capable of infecting plants, animals, and insects and causes the rotting of stored crops (Klich, 2007). It also produces aflatoxin B_1_ which has been classified as a class 1A carcinogen (Gizachew et al., 2019). Similarly, *Aspergillus niger* (*A. niger*) has been reported to be one of the most ubiquitous fungal species and possesses superior adaptability and survivability (Yu et al., 2021). *A. niger* is one of the fungi that dominate grain contamination in storage (Ju et al., 2020), and has been reported to cause black mold disease in onions, peanuts, and grapes (Ju et al., 2020). Additionally, *A. niger* has been recognized as having the potential to health issues in individuals with compromised immune systems (Hohl, 2017). Furthermore, *Fusarium oxysporum* (*F. oxysporum*) is also known for its negative effects on a wide range of plants, including crops, ornamental plants, and trees (Nag et al., 2022). It is responsible for causing wilt diseases in various plant species, including tomatoes, bananas, cucumbers, melons, and many others, and it produces toxins and enzymes that disrupt the water-conducting tissues (Maymon et al., 2020). The combating of the aforementioned fungal species often involves the use of synthetic pesticides which have been reviled due to their non-selective toxic effects on humans and others, as well as contaminating food chains through bioaccumulation in aquatic and terrestrial life (Lushchak et al., 2018). Consequently, the quest for the production of environmentally friendly anti-fungal agents is unwavering and EO from plants are gaining attention in this context.

*Callosobruchus maculatus* is a species of bruchid beetle that belongs to the family Chrysomelidae and cause severe damage to stored legume seeds including *Vigna unguiculata*, as well as other related crops like mung beans, black-eyed peas, and lentils. Thus, inflicting economic burdens on farmers in both developed and developing nations of the world (Allali et al., 2022). Consequently, there is an increasing demand for pesticides. However, it is well documented that the use of synthetic insecticides causes many diseases and pathological conditions to consumers of agricultural products, especially for agricultural workers. They cause neurological disorders, oxidative stress, DNA damage, metabolic disorders, and especially respiratory diseases (Kalpna et al., 2022). Hence, research aimed at developing safer alternatives to synthetic insecticides is unfaltering, and botanical extracts constitute one of the explored alternatives. Specifically, due to their low toxicity to mammals and their multiple modes of action, essential oils have been considered suitable alternatives (Cao et al., 2019). Interestingly, pesticides based on essential oils or their components have been shown to be effective against a wide range of agricultural and human disease vector pests (Radwan and Gad, 2021).

The antioxidant potentials of EO from medicinal plants are also well explored for the production of exogenous antioxidants for human consumption (Bouayed and Bohn, 2010). Notably, oxidative stress, which occurs as a result of an imbalance between the production of reactive oxygen species and their assuagement by the body’s antioxidant defense system, is known to play an active role in the pathogenesis and progression of diseases such as cancer, obesity, Alzheimer’s and cardiovascular diseases (Pizzino et al., 2017). Hence, there is a continued exploration of the EO of medicinal plants for their antioxidant potential.

*Haplophyllum tuberculatum* is a plant that has found numerous usage in folkloric medicine and has been reported to possess an array of pharmacological properties including anticancer, antioxidant, uterus-relaxing, antibacterial, and anti-HIV (Raissi et al., 2016). Extracts of *H. tuberculatum* are an important source of biologically active molecules, exemplifying this is the report by Agour et al. (2022) that, the aqueous extract of the aerial parts of *H. tuberculatum* promotes the healing of wounds, anti-inflammatory and analgesic activity. The extracts of this plant are also active against leishmaniasis (Hamdi et al., 2018; Mahmoud et al., 2020). Also, silver nanoparticles biosynthesized from *H. tuberculatum* extracts have strong antimicrobial activity against fecal and total coliforms. They also exert antibacterial power against clinically isolated Gram-negative and Gram-positive bacteria (El-Aswar et al., 2019). *H. tuberculatum* extracts also have a molluscicidal activity against *Physella acuta* (Benelli et al., 2015).

During biological activity, it is sometimes difficult, under the pressure of time or other factors, to elucidate the active compound present in EOs or plant extracts. Molecular docking is then necessary to provide rapid information on active compounds and their likely mechanism of action (Yusuf et al., 2022; Yusuf and Khan, 2023). In the coming years, *in silico* research is anticipated to assume a significant role across various domains of study, offering essential insights and enabling researchers to address intricate problems and make well-informed decisions (Yusuf, 2023).

However, despite the large number of studies that have been conducted on the biological activities of *H. tuberculatum*, the insecticidal power of the EO species remains much less studied. Furthermore, the exploration of its EO in the discovery of potent antifungals against the aforementioned fungal are mild. The formation of EO in plants is influenced by factors including geographical and climatic conditions, hence, their bioactive components tend to differ according to their source. Herein, this study explores the phytochemical constituents of HTEO and evaluates their insecticidal against *C. maculatus*, antioxidant activities, and antifungal activity against *A. flavu*s, *A. niger*, and *F. oxysporum* using *in silico* and *in vitro* techniques.

## 2 Materials and methods

### 2.1 Plant material

The aerial parts of *Haplophyllum tuberculatum* were systematically collected in February 2021 from a location near the city of Akka in southeastern Morocco. The geographical coordinates of the collection site were recorded as follows: Latitude: 29.39876053°N (29N 3252388.525 m N) and Longitude: 8.26860784°W (29N 570963.419 m E). The elevation of the collection site was measured at approximately 570,000 m. The taxonomic identification of the *H. tuberculatum* plant species was performed by a botanist from Sidi Mohamed Ben Abdellah University, Fez, Morocco. A reference sample of the collected plant material, labeled HT0019220211, was deposited in the herbarium of the Laboratory of Natural Substances, Pharmacology, Environment, Modeling, Health, and Quality of Life, Faculty of Sciences in Fez.

### 2.2 HTEO extraction

The essential oil of *Haplophyllum tuberculatum* was extracted using the hydrodistillation method with the Clevenger apparatus. 200 g of pre-dried aerial parts were placed in a 2 L flask, and 750 mL of water was added. Subsequently, the resulting mixture was left to boil for 3 h. The result HTEO obtained was stored at −4°C until utilization.

### 2.3 Chemical analysis of HTEO

#### 2.3.1 Gas chromatography analysis of volatile compounds in HTEO using flame ionization detection (FID)

The gas chromatograph utilized for this analysis was equipped with a flame ionization detector, and a CP-Sil-5CB Varian capillary column a CP-Sil-5CB Varian capillary column with dimensions of 50.00 m length, 0.32 mm internal diameter (id), and 1.25 µm thickness. The temperature program for the column was set to increase from 40°C to 280°C at a rate of 5°C per minute. During the analysis, the injector temperature was maintained at 250°C, while the detector temperature was set at 280°C to ensure optimal detection and quantification of the separated compounds. Prior to injection, the extracted HTEO was diluted in 10% hexane and 1 µL of it was injected into the gas chromatograph.

#### 2.3.2 Identification of chemical compounds in HTEO using gas chromatography-mass spectrometry (GC-MS)

The chemical compounds present in HTEO were identified using a Trace GC-ULTRA gas chromatograph coupled to a PolarisQ spectrometer (S/N-210729) with an ionization energy of 70 eV. The gas chromatograph was equipped with a TR5-CPSIL-5CB Varian capillary column, which had a diameter of 0.32 mm, a length of 50.0 m, and a film thickness of 1.25 µm. During the analysis, the column temperature program ranged from 40°C to 280°C, with a heating rate of 3°C per minute. The detector temperature was set to 200°C, and the injector temperature of the MS-PolarisQ was set to 260°C. Helium gas was utilized as the carrier gas with a fixed flow rate of 1 mL/min, and the HTEO was diluted in hexane. Ultimately, 1 µL of the diluted HTEO was injected into the gas chromatograph without fractionation using the injection mode. Noteworthy, the ion source temperature was maintained at 200°C, the interface temperature was set to 300°C, and the mass spectrometer operated in the scanning mode with a mass range of m/z 30–650. The volatile constituents of the essential oil were identified by determining the retention index (RI) of the EO compounds using a homologous series of n-alkanes, and comparing these calculated indices with the indices archived in the database (NIST MS Library v. 2.0; Adams, 2007).

### 2.4 Antifungal activity of HTEO

#### 2.4.1 Strains collection

Three mycelial strains *Aspergillus flavus, Aspergillus niger,* and *Fusarium oxysporum* (*A. flavus, A. niger,* and *F. oxysporum*) were utilized to evaluate the antifungal activity of *H. tuberculatum* EO. All strains were obtained from the Laboratory of Biotechnology, Environment, Agri-food, and Health, in the Faculty of Sciences Dhar El Mahraz, of the University of Sidi Mohammed Ben Abdellah, Fez, Morocco.

#### 2.4.2 Antifungal activity of HTEO by use of agar diffusion assay

The agar diffusion assay was adopted to evaluate the antifungal activity of HTEO (Balouiri et al., 2015). The three fungal strains were inoculated into Petri plates containing malt extract agar (MEA) medium. Sterile Whatman paper discs with a diameter of 6 mm were placed on the inoculated media surface and impregnated with 20 μL of *H. tuberculatum* EO (El Barnossi et al., 2020). The inoculated Petri plates were then incubated in the dark at 37°C. Ultimately, the percentage of inhibition was determined after 7 days using a negative control for each strain (Elegbede et al., 2019; El Barnossi et al., 2020). Statistical analysis was performed using Tukey’s test to compare the means of inhibition.

#### 2.4.3 Minimum inhibitory concentration (MIC) assay

The microdilution method was employed to determine the MIC of HTEO against the three fungal strains being studied (Balouiri et al., 2016). Each sterile microplate (96-well) was labeled, and then 0.1 mL of HTEO dissolved in 10% dimethyl sulfoxide (DMSO) was pipetted from the first well row to all other wells in the plate. Subsequently, serial dilutions of the HTEO were performed by transferring 50 µL of sterile malt extract (ME) into each consecutive well in the microplate using a multichannel pipette. Ultimately, 30 µL of the three fungal suspensions were added to each well. The inoculated microplates were then incubated for 7 days (Balouiri et al., 2016; Chebaibi et al., 2016). The determination of the MIC endpoint was performed by direct observation of microbial growth in the microplate wells. Also, a colorimetric test utilizing 0.2% (w/v) triphenyl tetrazolium chloride (TTC) was employed (Eloff, 1998; Dutra et al., 2016). Tukey’s test was employed to compare the means of the different treatment groups.

### 2.5 *In silico* evaluation of antifungal, and antioxidant activities of EOHT

The mechanism of action of phytochemicals identified in HTEO was theoretically investigated by use of *in silico* approach. To this end, the interaction of chemicals with the FAD glucose dehydrogenase and beta-1,4-endoglucanase from *A. flavus* and *A. niger* respectively was evaluated (Eno et al., 2022; Rased et al., 2022). While the NAD(P)H Oxidase was used to investigate the mechanism of action of the concerned chemicals as antioxidant agents.

The structures of the chemical compounds identified from HTEO were retrieved from the PubChem database (https://pubchem.ncbi.nlm.nih.gov/) in structure data format (SDF) (Kim et al., 2023). Subsequently, the structures were imported to the workspace of the Schrödinger Maestro 11.5 version before being prepared using the LigPrep tool with the OPLS3 force field. Preparatory steps taken include the generation of a maximum of 32 stereoisomers and the selection of ionization states at pH 7.0 ± 2.0.

Similarly, the crystal structures of the proteins FAD glucose dehydrogenase, a beta-1,4-endoglucanase, NAD(P)H Oxidase, were retrieved in protein databank (PDB) format from the PDB (https://www.rcsb.org/) (Berman, 2000) using the PDB IDs: 4YNT, 5I77, 2CDU.

The protein preparation wizard was utilized to prepare the protein as follows: the refinement of the structure, assignment of charges and bond orders, addition of hydrogens to heavy atoms, conversion of selenomethionines to methionines, and deletion of all water molecules. Noteworthy, the OPLS3 forcefield was employed to perform the minimization of the structures using the OPLS3e force field with the root mean square deviation (RMSD) value of heavy atoms set to 3.0. Ultimately, the receptor grid generation tool was employed to delineate the binding pocket of the protein with a volumetric spacing of 20 × 20 × 20.

### 2.6 Insecticidal activity against *C. maculatus*

The insecticidal activity of *H. tuberculatum* EO was assessed against *C. maculatus*. The insects utilized were obtained from a sample of *Cicer arietinum* (chickpea) from a stock in the region of Fez-Meknes. To maintain a sufficient number of insects for the experiments, mass rearing was conducted in glass jars. The glass jars were placed in a controlled environment with specific conditions including the photoperiod was set to 10 h of darkness and 14 h of light, mimicking natural light cycles, the relative humidity was maintained at a saturated level of 65% ± 5%, and the temperature was kept at a constant 25°C.

#### 2.6.1 Toxicity by contact

To assess the insecticidal activity of HTEO against *C. maculatus*, 100 g of chickpea seeds were infested with 10 individuals (five males and five females) of the *C. maculatus* for 2 days. The infested seeds were placed in lidded containers covered with a smooth, transparent cloth. To test the efficacy of HTEO as an insecticide, different concentrations of HTEO (1, 5, 10, and 20 μL/100 g) were added to separate containers containing the infested chickpea seeds, and the containers were manually shaken for 2 min to ensure uniform distribution of the oil. Additionally, a control group was established using five pairs of insects housed under the same conditions but without the addition of HTEO. Subsequently, the mortality of adult insects was assessed after 1 day of containment (Dutra et al., 2016). The count of eggs laid in the chickpea seeds was performed after 12 days of confinement, while the emerging insects were regularly counted after the first 28 days of confinement. Abbott’s formula below (Abbott, 1925), was utilized to correct for the observed mortality rate:

$$Pc=100\times \frac{P0-Pt}{100-Pt}$$

Where Pc and Po represent the corrected mortality and the observed mortality in the trial respectively, while Pt was the observed mortality in the control.

The reduction (%) in adults and eggs at each concentration of HTEO was calculated relative to the control using the following formula below [1]:

$$\mathrm{PR}=\frac{NC-NT}{CN}\times 100$$

Where PR (%) represents the percent oviposition or reduction of emerged insects, while NC and NT were the number of eggs or insects hatched in the control and treatment, respectively.

#### 2.6.2 Toxicity by inhalation

To perform this experiment, glass jars with a volume of 1,000 mL were used and small pieces of cotton were suspended inside the jars using a thread that was glued to the lid. Subsequently, different concentrations of HTEO (1, 5, 10, and 20 μL/1L of air) were deposited onto the cotton pieces, after which 10 *C. maculatus* insects, consisting of five males and five females with ages ranging from 0 to 2 days, were introduced into each jar, and completely sealed. Three replicates were performed for each concentration of EO. A control group, which consisted of cotton without EO, was also included for comparison purposes. To analyze the results, the observed mortality rate was corrected using the Abbott formula described in 1925 and was previously used in the contact test (Abbott, 1925).

#### 2.6.3 Repulsion test

The preferential surface area technique on filter paper was used to assess the repellent properties of HTEO (McDonald et al., 1970). The procedure involved using filter paper discs with a diameter of 90 mm, which was divided into two-halves. Subsequently, one of the halves of the filter paper disc was impregnated with a volume of 0.5 mL of HTEO diluted in acetone at various concentrations. The concentrations used were 0.016, 0.079, 0.157, and 0.315 μL/cm^2^ per disc. The other half of the disc was utilised as the control and was impregnated with 0.5 mL of acetone alone. After the 30 min exposure period, the number of *C. maculatus* insects present on the EO-treated half of the disk was counted and the number on the acetone-treated portion (control area) was also determined.

To calculate the percentage of repulsion (PR), the following formula was used (Zandi-Sohani et al., 2013):

$$\mathrm{PR}=\frac{NC-NT}{NC+NT}\times 100$$

Where PR represents the percentage of repulsion (%); NC and NT were the numbers of insects in the control and treatment areas respectively.

### 2.7 Evaluation of antioxidant activity

The antioxidant power of EOHT was evaluated using three tests, namely, the free radical scavenging DPPH assay, according to the protocol described in the study of Mssillou et al. (2021). The ferric reducing antioxidant power (FRAP) assay, according to the protocol described in the study of El Abdali et al. (2023); the measurement of total antioxidant capacity (TAC) according to the protocol reported in the study of Allali et al. (2021).

### 2.8 Data analysis

The mortality rate of *C. maculatus* insect was calculated using the Abbott formula (Balouiri et al., 2015). The analysis of variance (ANOVA) of repeated measures regarding the percentage of mortality by toxicity over time was calculated for 24, 48, 72, and 96 h. The determination of LC_50_ concentrations was done using the probit method (Hewlett, 1972) by the “IBM SPSS Program Version 21” software.

## 3 Results and discussion

### 3.1 Essential oil yield and GC-MS analysis

The yield of HTEO extracted by hydrodistillation was found to be 0.27%. However, it is worth noting that the yield of EO from *H. tuberculatum* can vary significantly and is influenced by various factors including maturity stage, genetic factors, environmental conditions, and geographic variations between habitats. This has also been observed as a phenomenon common with most aromatic and medicinal plant species. Generally, previous studies in which HTEO was extracted reported a yield that varied between 0.101% and 0.65% (Al-Burtamani et al., 2005; El-naggar et al., 2014; Bergheul et al., 2017; Sriti et al., 2017; Agour et al., 2020).

**FIGURE 1:**
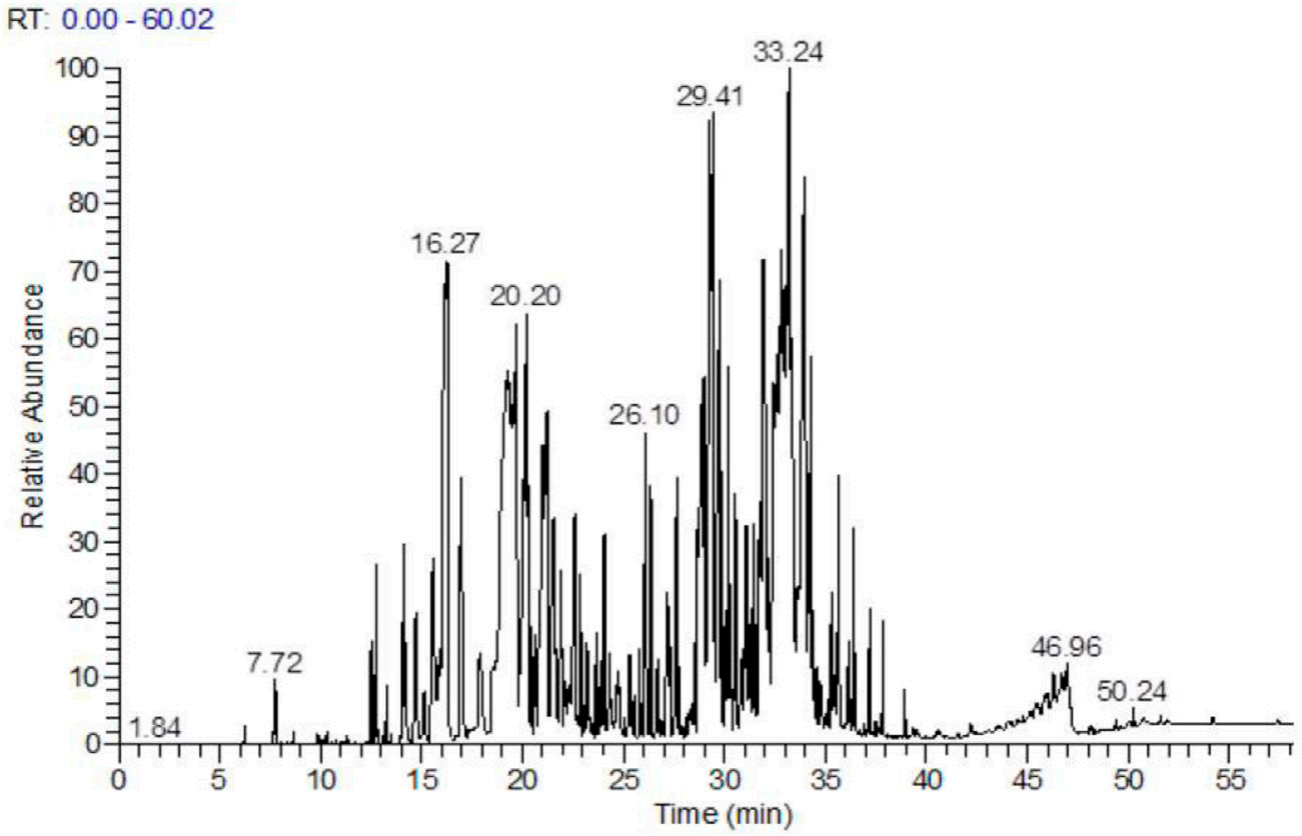
Chromatogram of HTEO.

Following the extraction of HTEO, GC-MS was employed to identify the phytochemical constituent, and the chromatogram whose peaks represent the compounds present is depicted in Figure 1. The results of the analysis revealed 34 compounds which are presented in Table 1. Analysis of the results revealed sesquiterpenes to be the most abundant class of compounds, accounting for 55.70% of the total composition. Monoterpenes were found to be the second most abundant at 25.83%, while diterpenes were present in very low quantities. The majority compound of the studied essential oil is *β*-Caryophyllene, a sesquiterpene molecule with a percentage of (36.94%), followed by monoterpenes, *α*-Phellandrene (14.72%), Germacrene D (8.72%) and Eugenol (2.49%).

**TABLE 1:** Chemical composition of HTEO obtained by GC-FID-MS.

Retention time (RT)	Compound name	Retention index (RI)		Chemical formula	Chemical class	Area (%)
		Cal	Lit			
12.77	*γ*-Terpinen	1,055	1,059	C_10_H_16_	Monoterpene (MO)	0.29
14.13	*β*-Guaiene	1,008	1,011	C_10_H_16_	MO	1.14
14.71	1,8-cineole	1,030	1,031	C_10_H_18_O	MO	1.91
15.55	p-Mentha-1,4 (8) diene	1,084	1,088	C_10_H_16_	MO	1.6
16.95	2-Carene	1,006	1,002	C_10_H_16_	MO	1.86
17.87	*α*-Guaiene	1,436	1,439	C_15_H_24_	Sesquiterpene (ST)	0.08
21.53	*α*-Terpinyl isobutyrate	1,471	1,473	C_14_H_24_O_2_	Others (O)	0.52
21.92	HexyI 2-methyl-3-pentenoate	1,320	1,322	C_12_H_22_O_2_	O	0.89
22.32	p-Mentha-7,8-dien-2-ol	1,189	1,189	C_10_H_16_O	MO	0.92
22.86	2-phenyl propanal	1,100	1,102	C_9_H_10_O	O	0.75
23.19	*Cis*-Verbenyl acetate	1,280	1,282	C_12_H_18_O_2_	O	0.10
24.04	Myrtenyl acetate	1,324	1,326	C_12_H_18_O_2_	O	0.23
24.31	p-Cymen-7-ol	1,287	1,290	C_10_H_14_O	MO	0.69
24.74	Carvacrol	1,297	1,299	C_10_H_14_O	MO	0.21
26.10	Eugenol	1,356	1,359	C_10_H_12_O_2_	MO	2.49
26.35	*γ*-Cadinene	1,510	1,513	C_15_H_24_	ST	0.13
26.68	*β*-sesquiphellandrene	1,520	1,522	C_15_H_24_	ST	0.65
27.27	*α*-Longipinene	1,350	1,352	C_15_H_24_	ST	0.28
27.63	*α*-Longifolene	1,388	1,390	C_15_H_24_	ST	1.14
29.26	β-Gurjunene	1,430	1,433	C_15_H_24_	ST	0.11
29.41	*α*-Phellandrene	1,001	1,002	C_10_H_16_	MO	14.72
29.75	*α*-Gurjunene	1,405	1,409	C_15_H_24_	ST	0.60
30.21	*α* -Cadinene	1,534	1,538	C_15_H_24_	ST	0.35
30.53	*α*-Cadinol	1,650	1,654	C_15_H_26_O	ST	0.86
31.08	Aristolochene	1,485	1,488	C_15_H_24_	ST	0.32
31.47	*cis-β*-Guaiene	1,490	1,493	C_15_H_24_	ST	0.77
31.92	Spathulenol	1,575	1,578	C_15_H_24_O	ST	0.15
32.44	Eudesm-7(11)-en-4-ol	1,700	1,700	C_15_H_26_O	ST	1.83
32.84	a-Selinene	1,494	1,498	C_15_H_24_	ST	0.20
33.24	*β*-Caryophyllene	1,408	1,408	C_15_H_24_	ST	36.94
34.30	Germacrene D	1,482	1,485	C_15_H_24_	ST	8.72
35.31	*α*-Bisabolol	1,684	1,685	C_15_H_26_O	ST	0.78
36.14	*β*-Bisabolol	1,673	1,675	C_15_H_26_O	ST	0.69
36.42	Viridiflorol	1,590	1,592	C_15_H_26_O	ST	0.37
		Monoterpene (MO)				25.83%
		Sesquiterpene (ST)				55.70%
		Others (O)				1.97%
		Total				83.50%

However, the phytochemical profile of HTEO is characterized mainly by the presence of oxygenated monoterpenes, monoterpene hydrocarbons, and non-terpene hydrocarbons, as well as oxygenated sesquiterpenes and sesquiterpene hydrocarbons (Mohammadhosseini et al., 2021). The main compounds, of the essential oils of *H. tuberculatum*, reported in previous studies were found to be different to those obtained in this study. According to (Hamdi et al., 2018), chromatographic analysis of the essential oil of *H. tuberculatum* revealed the presence of oxygenated monoterpenes (46.7%), monoterpene hydrocarbons (20.7%), and sesquiterpene hydrocarbons (0.5%). This variation could be attributed to the effect of geographical and environmental factors, as they not only influence the yield of EO but also the composition of the extracted oils. Table 2 presents some examples of the major compounds in HTEO reported in the literature.

**TABLE 2:** Major compounds of HTEO reported in literature.

Chemical composition of *H. tuberculatum* essential oils	Reference
*cis*-sabinene	Saad et al. (2022)
*trans*-*p*-menth-2-en-1-ol; *β*-phellandrene; piperitone and *cis*-p-menth-2-en-1-ol	Sriti et al. (2017)
*trans-p*-menth-2-en-1-ol; *cis*- and *trans-p*-menth-2-en-1-ol and piperitone	Hamdi et al. (2017b)
Limonene; *α*-pinene; *β*-pinene; *α*-phellandrene; *β*-phellandrene; myrcene, *δ*-3-carene; *β*-ocimene and *α*-terpinene	Mohamed Mohamed Sabry et al. (2015)
Linalool; linalyl acetate; 1,8-cineole and 4- terpineol	Mohamed Mohamed Sabry et al. (2015)
3-carene; *cis-p*-Menth-2- en-1-ol and *trans*- p-Menth-2- en-1-ol	El-naggar et al. (2014)
β- and γ-terpinene	Javidnia et al. (2006)
*α*-phellandrene	Al Yousuf et al. (2005)
limonene and α-pinene	Yari et al. (2000)

### 3.2 Antifungal activity

**FIGURE 2:**
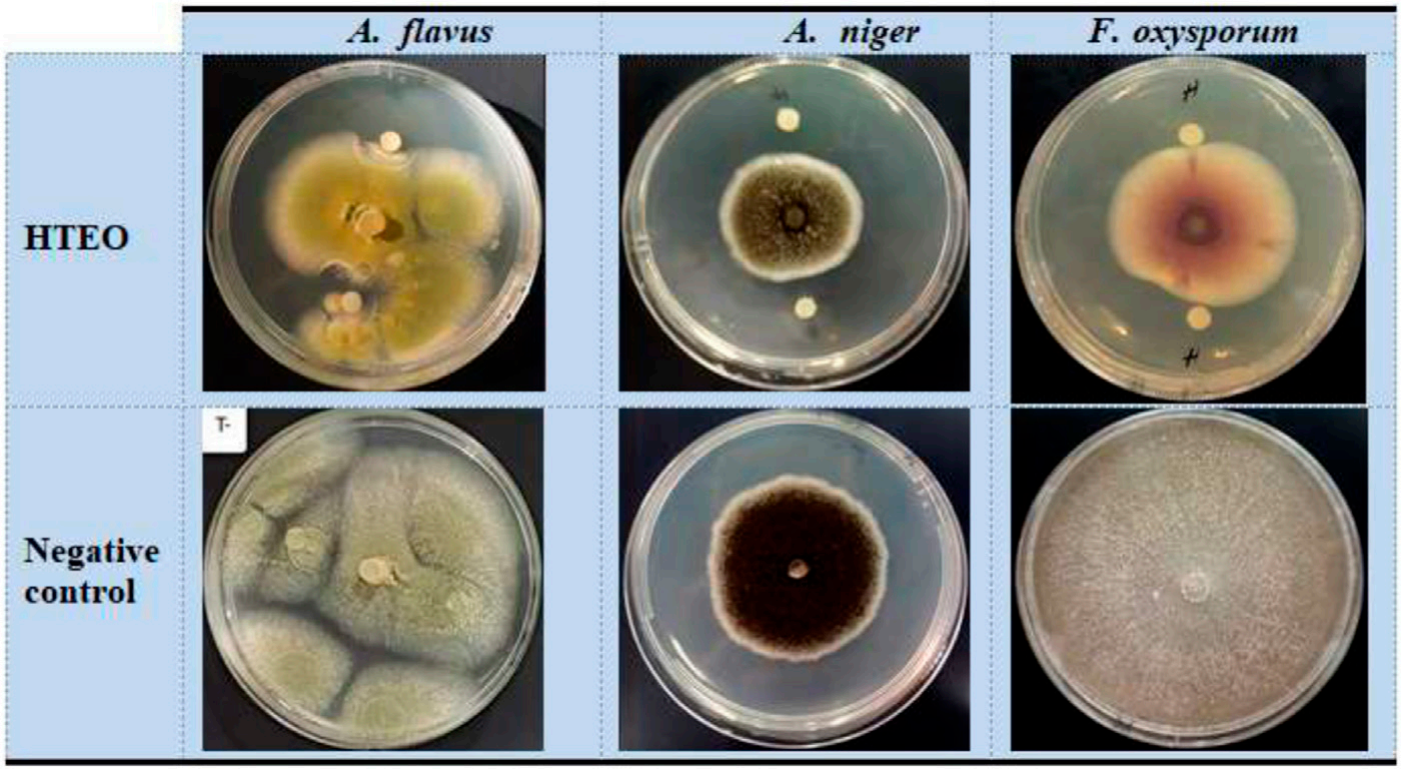
Antifungal activity of HTEO by use of the disc method.

The results of the evaluation of the antifungal activity of HTEO are presented in Table 3. HTEO exhibited inhibitory activity against all three fungal strains tested. Among the strains, *A. flavus* showed the highest sensitivity to HTEO, with an inhibition rate of 76.50% ± 0.60%, while *A. niger* exhibited moderate sensitivity with an inhibition rate of 61.22% ± 2.11%. *F. oxysporum* was found to be less sensitive to HTEO, with an inhibition percentage of 28.51% ± 0.69% (Figure 2). The MIC values, which indicate the minimum concentration required to inhibit the growth of the fungal strains, ranged from 20.53 ± 5.08 to 76.26 ± 5.08 mg/mL. The results of this study show that HTEO has limited antifungal efficacy against pathogenic molds.

**TABLE 3:** Antifungal activity of HTEO.

	% Inhibition	MCI (mg/mL)
*A. niger*	61.22 ± 2.11^a^	76.26 ± 5.08^a^
*A. flavus*	76.50 ± 0.60^b^	64.53 ± 5.08^a^
*F. oxysporum*	28.51 ± 0.69^c^	50.40 ± 5.54^b^

Values in the same column sharing different letters above are significantly different at *p* < 0.05.

Previous studies have also explored the antimicrobial properties of *H. tuberculatum* extracts. Exemplifying this is the results of the study by Abdelhhalek et al. (2020), in which they reported that the ethanolic extract of *H. tuberculatum* demonstrated inhibition rates of 82.96% and 93.70% against *F. culmorum* and *Rhizoctonia solani*, respectively. Additionally, two alkaloids, skimmianine and vulcanine, isolated from the aerial parts of *H. tuberculatum* exhibited antimicrobial effects against phytopathogenic bacteria and fungi. Skimmianine showed potent inhibitory activity against *Rhizobium radiobacter, Ralstonia solanacerum,* and *Pectobacterium carotovorum* ss*. carotovorum*, with a MIC of 62.5 mg/mL. In the antifungal assay, both skimmianine and vulcanine demonstrated inhibitory effects against *Verticillium dahliae, F. oxysporum,* and *Alternaria solani* (Abdelgaleil et al., 2020).

The antifungal effect of HTEO *on C. albicans* varies depending on the phytochemical composition of the extract and the specific strain of the fungal used. Hamdi et al. (2017a), reported that HTEO, had a MIC of 0.31 mg/mL against *C. albicans* ATCC 90028. In another study, the pure oil partially inhibited the growth of *C. albicans* ATCC 10231 with a diameter of 17.6 ± 0.3 mm (Al-Burtamani et al., 2005). Conversely, Sabry and El Sayed (2016) reported that essential oils obtained from the aerial parts of *H. tuberculatum* were inactive against *C. albicans* strain CBDN 05036. However, HTEO was also reported to exert antimycelial activity in addition to its antifungal activity. Furthermore, Al-Burtamani et al. (2005) also reported that HTEO inhibited the growth of *F. oxysporum* and *A. flavus* with MIC values lower than 1 mg/mL.

### 3.3 *In-silico* evaluation of the antifungal, antioxidant and activities of HTEO

Following the docking of the phytochemicals identified in HTEO against the previously mentioned targets, their binding affinities for the proteins as revealed by their docking score presented in Table 4, were assessed. For antifungal activity, *γ*-Cadinene and p-Cymen-7-ol with docking scores of −6.322 and −6.302 kcal/mol, respectively, were found to possess the highest affinities for *A. flavus* FAD glucose dehydrogenase while Carvacrol and Viridiflorol also had the highest affinities for *β*-1,4-endoglucanase from *A. niger* with docking scores of −5.647 kcal/mol and −5.453 kcal/mol. In antioxidant activity, *α*-Terpinyl isobutyrate showed high affinity for the active site of NADPH oxidase with a glide score of −6.188 kcal/mol.

**TABLE 4:** Docking results with ligands in different receptors.

	Glide gscore (Kcal/mol)		
	2CDU	4YNT	5I77
1,8-cineole	−4.399	−5.253	−4.048
2-Carene	−4.53	−5.175	−3.958
*α*-Bisabolol	−5.468	−5.604	−3.892
*α*-Cadinene	−5.432	−5.294	−4.255
*α*-Guaiene	−4.555	−5.122	−3.875
*α*-Gurjunene	−5.167	−6.15	−4.294
*α*-Longifolene	−4.704	−5.504	−4.018
*α*-Longipinene	−5.195	−5.501	−4.468
*α*-Phellandrene	−4.693	−5.339	−4.358
*α*-Selinene	−4.81	−5.768	−3.631
*α*-Terpinyl isobutyrate	−6.188	−5.478	−3.433
Aristolochene	−4.564	−4.773	−3.77
*β*-Bisabolol	−4.072	−5.352	−3.634
*β*-Caryophyllene	−4.675	−5.591	−3.708
*β*-Guaiene	−4.778	−6.301	−3.831
*β*-Gurjunene	−5.11	−5.927	−3.912
*β*-sesquiphellandrene	−3.343	−4.48	−2.449
Carvacrol	−6.027	−6.17	−5.647
*cis*-*β*-Guaiene	−4.323	−5.475	−3.743
*Cis*-Verbenyl acetate	−4.789	−5.336	−3.964
Eudesm-7(11)-en-4-ol	−4.68	−5.592	−4.775
Eugenol	−4.422	−4.646	−3.754
*γ*-Cadinene	−5.447	−6.322	−4.436
*γ*-Terpinene	−5.12	−5.54	−3.945
Germacrene D	−5.085	−5.46	−4.054
Myrtenyl acetate	−4.787	−5.947	−3.705
p-Cymen-7-ol	−5.291	−6.302	−4.263
p-Mentha-1,4 (8)-diene	−5.066	−5.125	−3.749
Spathulenol	−5.126	−5.674	−4.72
Viridiflorol	−4.397	−5.556	−5.453

**FIGURE 3:**
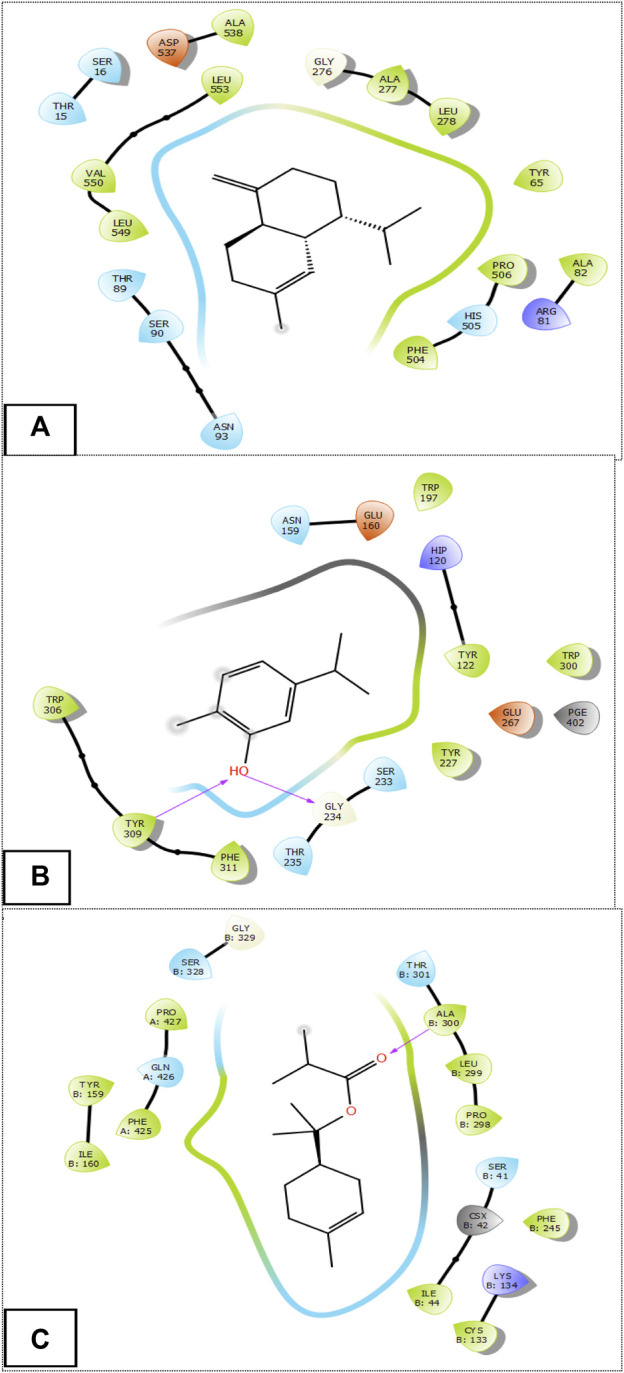
The 2D viewer of ligands interactions with the active site. **(A)**: *γ*-Cadinene interactions with active site of *A. flavus* FAD glucose dehydrogenase. **(B)**: Carvacrol interactions with active site of *α*-1,4-endoglucanase from *A. niger*. **(C)**: *α*-Terpinyl isobutyrate interactions with active site of NADPH oxidase.

**FIGURE 4:**
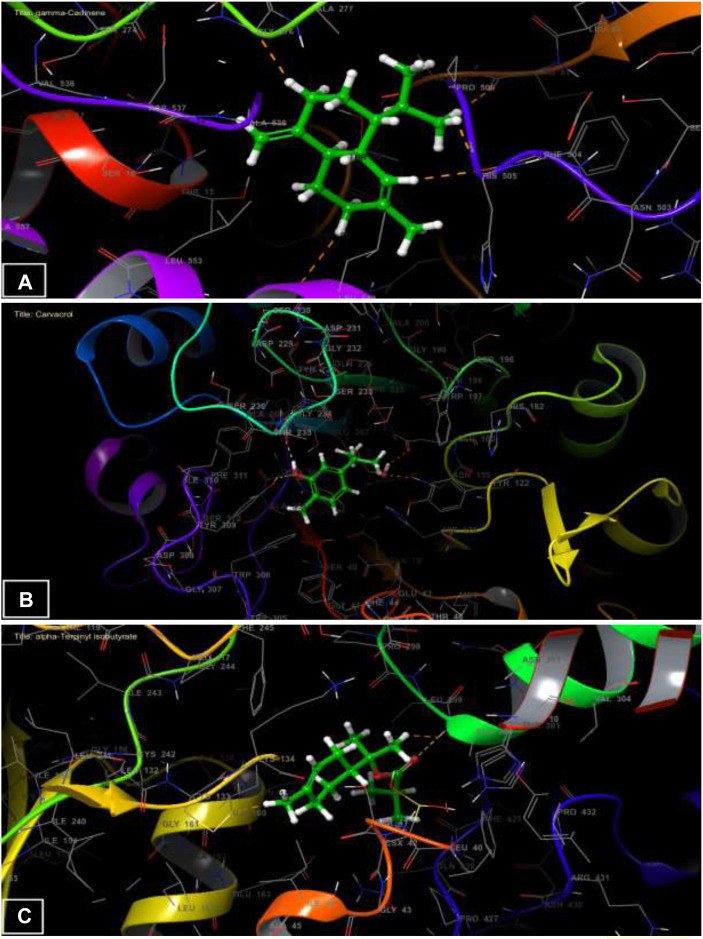
The 3D viewer of ligands interactions with the active site. **(A)** *γ*-Cadinene interactions with active site of *A. flavus* FAD glucose dehydrogenase. **(B)** Carvacrol interactions with active site of *β*-1,4-endoglucanase from *A. niger.* **(C)** *α*-Terpinyl isobutyrate interactions with active site of NADPH oxidase.

The Figure 3 and Figure 4 represent the 2D viewer and the 3D viewer of ligands interactions with the active sites, respectively. Analysis of the resulting complexes to delineate the interactions between them revealed that Carvacrol interacted with the active site amino acid residues of *A. niger β*-1,4-endoglucanase via two hydrogen bonds with residues including TYR 309 and GLY 234. In the antioxidant activity, *α*-Terpinyl isobutyrate established one hydrogen bonds in the active site of NADPH oxidase with residues ALA 300.

Few studies report on the *in silico* activities of the major compounds in the essential oil studied. However, studies related to the activities described in the present work have been identified. A previous study reported that *trans*-caryophyllene and eugenol had a more stable binding strength in the acetylcholinesterase enzyme of *Tribolium castaneum*, and there was synergy between eugenol and *trans*-caryophyllene when the two compounds interacted with acetylcholinesterase (Ikawati et al., 2022). On the other hand, the *in silico* study of the anti-gout and anti-diabetic activities of the essential oil from *Piper lolot* reveals that the major compound, *β*-caryophyllene (20.6%), exhibits inhibitory effects against the enzymes *α*-glucosidase, *α*-amylase and xanthine oxidase, with docking scores of −7.4, −6.2, and −5.8 kcal/mol, respectively (Nguyen et al., 2023).

The *in silico* study of the antioxidant activity of compounds in *Juniperus thurifera* bark essential oil shows that *α*-cadinol and muurolol have inhibitory activity on the active site of NADPH oxidase with a glide score of −6.041 and −5.956 kcal/mol, respectively (Lafraxo et al., 2022). Similarly, *β*-terpineol, carvacrol, and thymol, contained in *Lavandula dentata* essential oil were found to be active against NADPH oxidase, with a displacement score of −4.728, −6.17, and −6.483 kcal/mol, respectively (El Abdali et al., 2023).

### 3.4 Insecticidal activity

**FIGURE 5:**
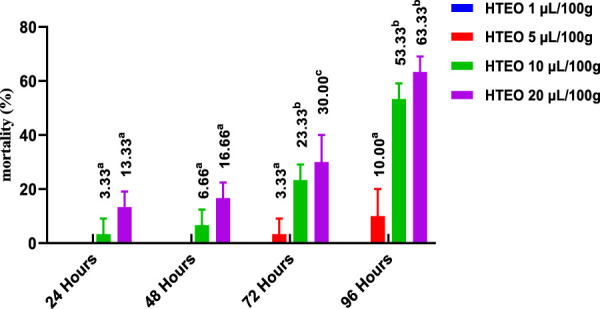
Effects of HTEO tested by Inhalation on the mortality of the adults of the bruche *C. maculatus.* Tukey test: a different letter on the same row indicates a significant difference (*p* < 0.05).

**FIGURE 6:**
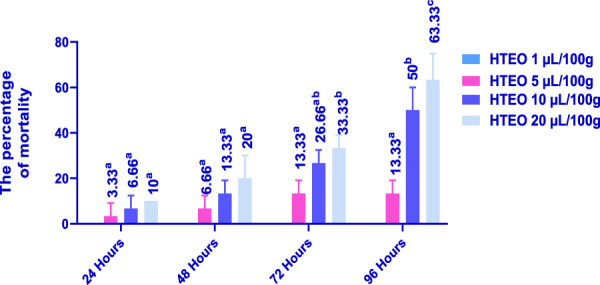
Effects of HTEO on *C. maculatus* by contact test. In the insecticidal activity all the tested doses were compared with the negative control (0 μL). Tukey test: a different letter on the same row indicates a significant difference (*p* < 0.05).

The insecticidal activity of HTEO was assessed via inhalation and contact against adults of *C. maculatus* and the results of the study are presented in Figures 5, 6. The inhalation test showed that after 24 h, only the doses of 10 and 20 μL/100 g resulted in low percentages of mortality in *C. maculatus* adults, with 3.33% and 13.33% respectively. After 96 h, the 1 μL/100 g dose still had no effect, while the doses of 10 and 20 μL/100 g resulted in mortality percentages above 50%. The contact mortality test showed similar results, with the concentration of 5 μL/100 g causing 3.33% mortality after 24 h and 13.33% after 96 h. The highest dose (20 μL/100 g) resulted in 10% mortality after 24 h and 63.33% after 96 h. Overall, the contact toxicity of HTEO was higher against *C. maculatus* through the contact test compared to the inhalation test.

The LC_50_ and LC_95_ values obtained from the contact test were lower than those obtained from the inhalation test. After 24 h, the contact test yielded LC_50_ values of 30.66 and 40.28 μL/100g, while the inhalation test resulted in LC_50_ values of 14.59 and 14.68 μL/100 g. After 96 h, the LC_50_ values were 27.95 and 28.40 μL/100 g for the contact and inhalation tests, respectively.

**FIGURE 7:**
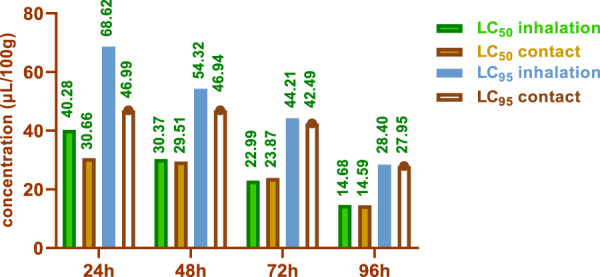
Lethal concentrations of HTEO against *C. maculatus*.

The LC_50_ and LC_95_ values obtained from the contact test were lower than those obtained from the inhalation test (Figure 7). Notably, after 24 h, the contact test yielded LC_50_ values of 30.66 and 40.28 μL/100g, while the inhalation test resulted in LC_50_ values of 14.59 and 14.68 μL/100 g. After 96 h, the LC_50_ values were 27.95 and 28.40 μL/100 g for the contact and inhalation tests, respectively.

**FIGURE 8:**
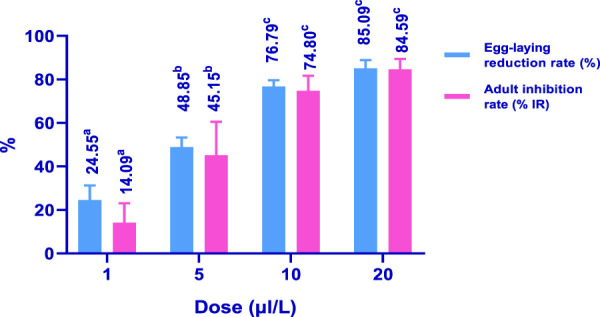
Effects of HTEO on the emergence and fecundity of *C. maculatus*. Values of the same parameter sharing different letters above the bars are significantly different at *p* < 0.05.

In the control group, *C. maculatus* bruchid females laid 207.33 eggs. Interestingly, the use of 20 μL/100 g of HTEO significantly reduced the emergence of bruchids by 91.34%. As depicted in Figure 8, HTEO significantly reduced the fecundity of *C. maculatus*. Specifically, the concentration of 5 μL/100 g resulted in a remarkable reduction in oviposition rate (48.85%) and emergence inhibition (45.15%) compared to the negative control. The concentration of 20 μL/100 g induced a significant reduction in oviposition rate (85.09%) and emergence inhibition (84.59%). 207.33 was the number of eggs laid by *C. maculatus* bruchid females, and the use of 20 μL/100g, significantly reduced the emergence with a rate of 91.34%. The results presented in Figure 8 show that HTEO significantly reduced fecundity in the insect pest *C. maculatus*. The concentration 5 μL/100g, resulted in a very remarkable rate of oviposition reduction (48.85%), and emergence inhibition (45.15%) compared to the negative control. on the other hand, the concentration of 20 μL/100 g induces a very important rate of oviposition reduction and emergence inhibition, which are respectively 85.09% and 84.59%.

### 3.5 Repulsive activity

**FIGURE 9:**
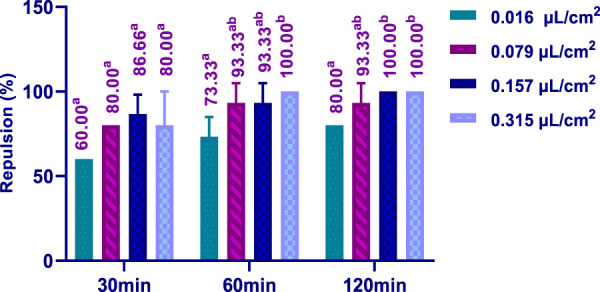
Repellent activity of *H. tuberculatum* EOs against insects of *C. maculatus* Experiments were performed with replicates (3 times). p: One-way ANOVA was adopted in determining of significant differences. Tukey test: a different letter on the same row indicates a significant difference (*p* < 0.05).

The repellent effect of HTEO against the insect *C. maculatus* was assessed using the preferential surface method on filter paper, and the results obtained are depicted in Figure 9. It was observed that the repellent activity was dose-dependent. At the lowest concentration tested (0.016 μL/cm^2^), a repulsion rate of 60% was observed after 30 min, which further increased to 80% after 120 min of application. The maximum repellent effect *against C. maculatus* was achieved after 60 min by using a concentration of 0.315 μL/cm^2^. These findings highlight the potent repellent properties of HTEO against *C. maculatus*, hence, rendering it worthy of further exploration.

To the best of our knowledge, this study is the first to investigate the insecticidal activity of HTEO against *C. maculatus*. To this end, the determination of toxicity by contact and inhalation test shows significant results with LC_50_ values of 14.59 and 14.68 µL/100 g, respectively. Previous studies have been conducted on the insecticidal potentials of HTEO against other insect pests, as well as the insecticidal activities of extracts of plants belonging to the same genus or family as *H. tuberculatum* (Rutaceae) against *C. maculatus* using different protocols including fumigation, repellent and contact (Mssillou et al., 2022b).

Noteworthy, studies have shown that the insecticidal efficacy of EO extracted from various plant species varies against *C. maculatus*. In a study, the EO of a total of 121 plant species from 26 families were reported to possess insecticidal effects against *C. maculatus* (Mssillou et al., 2022b). Notably, in another study by Allali *et al.*, the EO of *Syzygium aromaticum*, primarily composed of 80.26% eugenol, exhibited insecticidal activity against *C. maculatus* in inhalation tests, causing mortality rates of 6.67% at 24 h, 36.67% at 48 h, and 100% at 72 h. This EO also reduced oviposition and completely suppressed adult emergence (Aimad et al., 2021). Similarly, the EO of *Dittrichia viscosa* L., which contained bornyl acetate as the most abundant compound with 41%, resulted in 60% mortality in the contact test and 97.5% mortality in the inhalation test after 96 h of exposure to a 1 µL dose (Mssillou et al., 2022a). Also, the application of 20 μL of the EO of *Dittrichia viscosa* L. on *C. maculatus*, causes an approximately 91% reduction in the number of eggs laid by this insect. In another study, the insecticidal activity of the EO of *Atalantia monophylla*, containing eugenol (19.76%) and sabinene (19.57%), against *C. maculatus* using the fumigation test was found to result in 70.22% mortality, 85.24% repellency test, and also exhibited ovicidal activity (Nattudurai et al., 2017). Additionally, essential oils extracted from three Citrus species demonstrated insecticidal effects against *C. maculatus*, with LC_50_ values of 6.33, 7.21, and 8.70 μL/L of air for *C. aurantium, C. limon,* and *C. reticulate*, respectively (Saeidi et al., 2011). It is also worth noting that HTEO has also been reported to possess insecticidal activities against other insect species infesting plant products. Exemplifying this is the contact toxicity showed by HTEO against the adults of three insect pests of cereals, namely, *Sitophilus oryzae, Tribolium castaneum* and *Trogoderma granarium* with LC_50_ values of 0.11, 0.048, and 0.13 mg/cm^2^, respectively (Saad et al., 2022). Additionally, HTEO extracted from aerial parts showed strong activity against *Aedes aegypti* larvae, specifically, the EO used had the potential to kill *Aedes aegypti* larvae at 125 ppm (Al-Rehaily et al., 2014).

Other extracts obtained from *H. tuberculatum* have also demonstrated insecticidal activities. The aqueous extract of *H. tuberculatum*, at concentrations of 5% and 10%, reduced egg hatching and juvenile mobility in *Meloidogyne javanica*, and showed a toxic effect on eggs and juveniles. At these same concentrations, the immobility of juveniles reached 100% after 48 h of exposure (Kallel et al., 2009). The methanolic extract of *H. tuberculatum* has also been reported to negatively impact the essential reproduction parameters of female *Locusta migratoria*. In this regard, the oral administration of this extract to newly emerged females (1.5 g/female), resulted in significant negative effects in terms of delay of the first oviposition, as well as reduction of fecundity and fertility (Acheuk et al., 2012).

Although the mode-of-action (MOA) of the observed HTEO-mediated antifungal and insecticidal activities remains to be elucidated and is still being explored, it is commonly believed that the complex mixture of compounds present in the EO might act synergistically, instigating several biological effects simultaneously. In this context, numerous studies have reported that the phytochemicals present in EOs can disrupt the cytoplasmic membrane and influence a range of other intracellular signaling and biological process, particularly energy generation (Swamy et al., 2016). Reduced membrane potentials, proton pump disruption, and ATP depletion have been reported as contributing factors to the reported antifungal activity (Turina et al., 2006; Chebbac et al., 2022).

Some reports have elaborated on the MOA of individual constituents present in EOs against insect pests. For example, a recent study revealed that the EO derived from *Mentha arvensis* exhibits a systemic MOA against *Sitophilus granarius*, and this was ascribed to the ability of the EO to perturb the insect’s neurological and muscular systems, intracellular aerobic respiratory processes, and cuticle, which serves as the primary protective barrier of insects (Renoz et al., 2022). Menthol and eugenol, both abundant in HTEO, have been shown to act on the octopaminergic system, triggering octopamine receptors and the phosphorylation pathway for protein kinase A in insect neurons (Hong et al., 2018; Jankowska et al., 2019). In *Aphis gossypii*, β-caryophyllene, another constituent enriched in HTEO, has been demonstrated to inhibit several enzymatic targets, including acetylcholine esterase, polyphenol oxidase, and carboxylesterase (Liu et al., 2010).

### 3.6 Antioxidant activity

The results of the antioxidant activity of HTEO obtained using three tests (DPPH, FRAP and TAC) are presented in Table 5. As evident from the table, HTEO demonstrates a significant antioxidant activity with a value of 758.34 ± 3.87 mg AAE/g of EO. This potent activity can be attributed to the oil’s high content of various terpene compounds. While the EC_50_ and IC_50_ values of EOHT were only slightly higher compared to those of the standard compounds, they still indicate strong antioxidant activity. A comparison of the antioxidant capacity to that of the aqueous and ethanolic extracts of other samples collected from the same region revealed a higher total antioxidant capacity. Also, the aqueous extract demonstrates lower free radical scavenging activity (IC_50_ = 0.37104 mg/mL) compared to the EO. However, both extracts show slightly higher iron-reducing activity (EC_50_ values of 0.15554 and 0.16944 mg/mL for the aqueous and ethanolic extracts, respectively) (Agour et al., 2022).

**TABLE 5:** results of the antioxidant activity (DPPH, FRAP and TAC).

	DPPH (IC_50_ mg/mL)	FRAP (EC_50_ mg/mL)	TAC (mg AAE/g ext)
EOHT	0.253	0.294	758.34 ± 3.87
Ascorbic acid	0.0027	0.000799	—
Quercetin	0.007295	0.007295	—

Similar findings have been reported in other studies investigating the antioxidant activity of EO extracted from the leaves and stems of *H. tuberculatum*. It showed moderate antiradical activity against DPPH (IC_50_ = 0.14 mg/mL) (Hamdi et al., 2017a). The results are consistent with a previous study by Debouba *et al.* (2014) (Debouba et al., 2014), which reported moderate antioxidant activity of *H. tuberculatum* oils against free radicals and low iron-reducing power. In an *in vivo test* on diabetic rats, the EO obtained from the flowers and aerial parts of *H. tuberculatum* significantly restored the reduced level of glutathione, indicating their antioxidant potential (Sabry and El Sayed, 2016). The antioxidant activities observed in the essential oils can be attributed to their chemical composition, particularly the high content of monoterpene compounds (25.83%). The antioxidant activities of essential oils are often associated with concepts such as additivity, antagonism, and synergy, as the presence of multiple compounds can contribute to the overall antioxidant capacity (Harkat-Madouri et al., 2015). Therefore, the high antioxidant activity of HTEO can be attributed to its chemical composition, particularly the abundance of monoterpene compounds.

## 4 Conclusion

Conclusively, the results of this study provide valuable insights into some biological activities of HTEO particularly against the infamous *C. maculatus* and also evaluate its potential to serve as a biocide for the control of the fungal species including *A. flavus*, *A. niger*, and *F. oxysporum*. Specifically, HTEO demonstrated insecticidal activity that surpasses that of EO derived from other plant species, while also exhibiting high total antioxidant capacity that is attributable to the presence of various terpene compounds. The results of assessing the antifungal activity of HTEO through molecular docking reveal the presence of compounds that could also potentially serve as a viable alternative to the synthetic pesticides commonly employed to combat pathogenic fungi in agricultural products. Overall, EOHT presents itself as a promising candidate for further development and utilization in pest management strategies and antioxidant interventions, promoting sustainable and eco-friendly alternatives in these domains.

## Data Availability

The raw data supporting the conclusion of this article will be made available by the authors, without undue reservation.
